# Exogenous IL-17A Alleviates Social Behavior Deficits and Increases Neurogenesis in a Murine Model of Autism Spectrum Disorders

**DOI:** 10.3390/ijms25010432

**Published:** 2023-12-28

**Authors:** Yehoshua Willinger, Daniella R. Friedland Cohen, Gadi Turgeman

**Affiliations:** 1 Department of Molecular Biology, Faculty of Natural Sciences, Ariel University, Ariel 40700, Israel; yehoshuawi@ariel.ac.il (Y.W.); mondaniella@gmail.com (D.R.F.C.); 2 The Adelson School of Medicine, Ariel University, Ariel 40700, Israel

**Keywords:** autism spectrum disorder (ASD), hippocampal neurogenesis, IL-17A, social behavior

## Abstract

Among the proposed mechanisms for autism spectrum disorders (ASD) is immune dysregulation. The proinflammatory cytokine Interleukine-17A (IL-17A) was shown to play a key role in mediating immune-related neurodevelopmental impairment of social behavior. Nevertheless, post-developmental administration of IL-17A was found to increase social behavior. In the present study, we explored the effect of post-developmental administration of IL-17A on ASD-like behaviors induced by developmental exposure to valproic acid (VPA) at postnatal day 4. At the age of seven weeks, VPA-exposed mice were intravenously injected twice with recombinant murine IL-17A (8 μg), and a week later, they were assessed for ASD-like behavior. IL-17A administration increased social behavior and alleviated the ASD-like phenotype. Behavioral changes were associated with increased serum levels of IL-17 and Th17-related cytokines. Exogenous IL-17A also increased neuritogenesis in the dendritic tree of doublecortin-expressing newly formed neurons in the dentate gyrus. Interestingly, the effect of IL-17A on neuritogenesis was more noticeable in females than in males, suggesting a sex-dependent effect of IL-17A. In conclusion, our study suggests a complex role for IL-17A in ASD. While contributing to its pathology at the developmental stage, IL-17 may also promote the alleviation of behavioral deficits post-developmentally by promoting neuritogenesis and synaptogenesis in the dentate gyrus.

## 1. Introduction

Autism spectrum disorders (ASD) are a collection of neurodevelopmental disorders characterized by repetitive and routinized behavior, social avoidance, and cognitive rigidity [1,2]. One out of 100 children will be diagnosed with ASD, and the prevalence is four times greater in males than in females. While the etiology of ASD is largely unknown, both genetic and epigenetic changes due to prenatal and early postnatal exposure to infections, stress, and chemicals are considered plausible causes [3,4,5,6]. For example, valproic acid (VPA), a fatty acid widely used as a neuropsychiatric drug [7], was shown in several studies to increase the risk for ASD in children exposed to the drug prenatally [8].

Several studies have also demonstrated widespread changes in immune function in people with ASD [9,10]. These changes were found to be related to impairments in core features in ASD, such as decreased adaptability and poor cognitive abilities [11]. Individuals with ASD seem to have an inflammatory profile, both in the central nervous system and in the periphery. Furthermore, there is a growing recognition for the role of immune dysregulation and in particular for the pro-inflammatory cytokine IL-17A in the etiology of ASD during development [12]. Nevertheless, although developmental exposure to IL-17A is associated with ASD development, recently it was shown that intracerebral administration of IL-17A at adulthood ameliorated social deficits inflicted by maternal immune activation [13].

Neurogenesis during adult life occurs mainly in the subgranular zone (SGZ) of the dentate gyrus (DG) of the hippocampus and the subventricular zone (SVZ) of the lateral ventricle [14,15]. During neurogenesis, newly formed neurons mature and undergo morphological changes by which membrane sprouts grow to form neurites in their dendritic and axonal poles, which are necessary to establish synapses, in a process termed neuritogenesis [16]. Newly formed neurons functionally integrate into the hippocampal network and are mechanistically involved in the process of learning and memory [15,17,18]. An association between hippocampal neurogenesis and behavior has been observed in many studies [18,19]. Impaired neurogenesis is associated with several neurobehavioral disorders [20,21,22], including autism spectrum disorders [23]. In particular, impaired neuritogenesis was suggested as a key feature in the pathophysiology and/or therapy of ASD [24,25]. In the VPA model for ASD, aberrant adult neurogenesis was observed as well. An increased number of newly formed neurons with a reduced neuroprogenitor pool were detected in the dentate gyrus [26,27,28].

The immune system can affect adult hippocampal neurogenesis in several ways, namely by the secretion of chemokines and cytokines and by the activation of immune cells [29,30]. We previously discovered that a single administration of interleukin IL-17A to adult naïve mice slightly improved spatial learning and altered neurogenesis by inhibiting neuroprogenitor proliferation while increasing neurite growth and neuronal maturation [31]. Similarly, we discovered that high levels of systemic IL-17A improved social interaction behavior in mice following exposure to trauma, which was associated with the expected changes in neurogenesis [32].

In the present study, we sought to explore the effect of IL-17A administration at adulthood on the ASD-like phenotype and neurogenesis in an established murine model of autism induced by developmental exposure to VPA [33]. We postulated that in contrast to developmental exposure, IL-17A administration at adulthood may correct alterations in neurogenesis induced by VPA and improve behavior, particularly social behavior, as we previously observed [32]. In addition, we explored the effect of short-term IL-17A administration on serum cytokine levels and whether sex-dependent differences were observed, as we previously reported [33,34]. Our results can shed light on the complex role of IL-17A in ASD, suggesting a protective role for it at adulthood. 

## 2. Results

### 2.1. Treatment with IL-17A Improves Social Behavior and the General ASD-Like Phenotype

**Figure 1:**
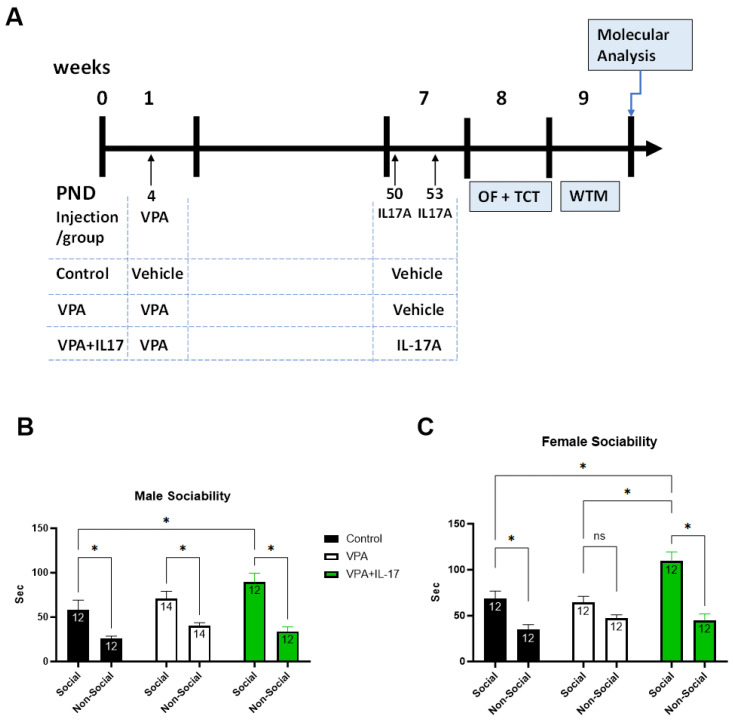
Administration of IL-17A improved social behavior in VPA mice. (**A**) A diagram outlining the experimental design according to experimental post-natal timeline. Time is marked in weeks above the timeline and in post-natal days (PND) below the timeline. Injection time points with VPA or IL-17A are marked and outlined for each treatment group. Control—represents control mice group not exposed to VPA and without any treatment. VPA—represents mice exposed to VPA modelling ASD without any treatment. VPA+IL-17—represents mice exposed to VPA that were treated with IL-17A at adulthood. OF—open field paradigm. TCT—three-chamber social interaction test paradigm. WTM—water T-maze paradigm. Social interaction was assessed in VPA-exposed mice w/o IL-17A treatment via the TCT paradigm. (**B**) A graph presenting the interaction duration in seconds of tested male mice with either a social stimulus (novel mouse) or a nonsocial stimulus (empty cage) for each group. (**C**) A graph presenting the interaction duration in seconds of tested female mice with either a social stimulus (novel mouse) or a nonsocial stimulus (empty cage) for each group. Data are presented as the mean ± SE and the number of samples per group is presented within the bars. * *p* < 0.05, ns—non significant, two-way ANOVA.

The ASD-like phenotype was induced in ICR mice by administering valproic acid (VPA, 300 mg/kg) subcutaneously at the age of four days, as we previously described [33]. Control animals were injected with a similar volume of 0.9% saline. At the age of seven weeks, IL-17A (8 µg/µL) was administered twice, intravenously, in a three-day interval into VPA-exposed mice. A week later, behavior assays were executed to evaluate ASD-like characteristics (Figure 1A). In the three-chamber social interaction (TCT) paradigm, IL-17A treatment increased social interaction duration in both males and females (Figure 1B,C). In females, VPA-exposed mice exhibited impaired preference for the social stimulus, which was corrected by IL-17A administration. Furthermore, IL-17A treatment increased social interaction duration compared with nontreated VPA-exposed mice and controls in females (Figure 1C). In males, we did not observe impaired preference toward social stimuli in VPA-exposed mice; nevertheless, IL-17A increased social interaction in VPA-exposed mice compared with controls (Figure 1B).

**Figure 2:**
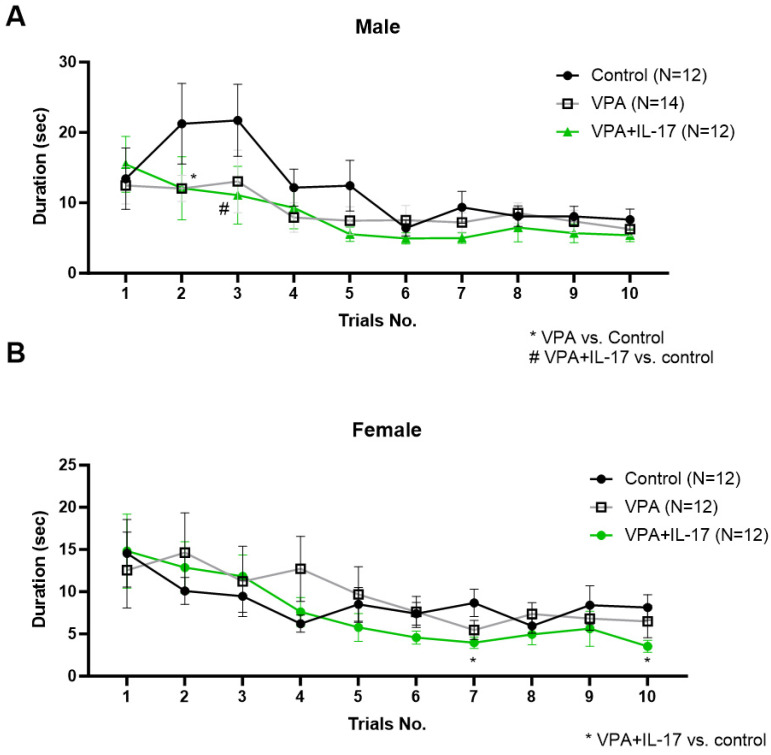
Administration of IL-17A improved cognitive rigidity in VPA mice. Cognitive rigidity was assessed using the water T-maze test at the age of 9 weeks. The latency duration in seconds to relocate the platform on the third day of the assay was measured in 10 successive trials in males (**A**) and females (**B**). Control—represents control mice group, not exposed to VPA and without any treatment. VPA—represents mice exposed to VPA modelling ASD without any treatment. VPA+IL-17—represents mice exposed to VPA that were treated with IL-17A at adulthood. Data are presented as the mean ± SE and number of samples per group is indicated in the graph legend. ^#,^* *p* < 0.05, two-way ANOVA repeated measurements.

Cognitive rigidity was assessed using the water T-maze paradigm. In this assay, mice learned the position of a platform in one arm of the maze for two days, and on the third day, the position of the platform was changed to the other arm. The time taken by the mice to relocate the platform was measured in 10 consecutive trials. IL-17A-treated animals displayed slightly shorter durations during some of the trials compared with one of the other groups, both in males and females (Figure 2).

**Figure 3:**
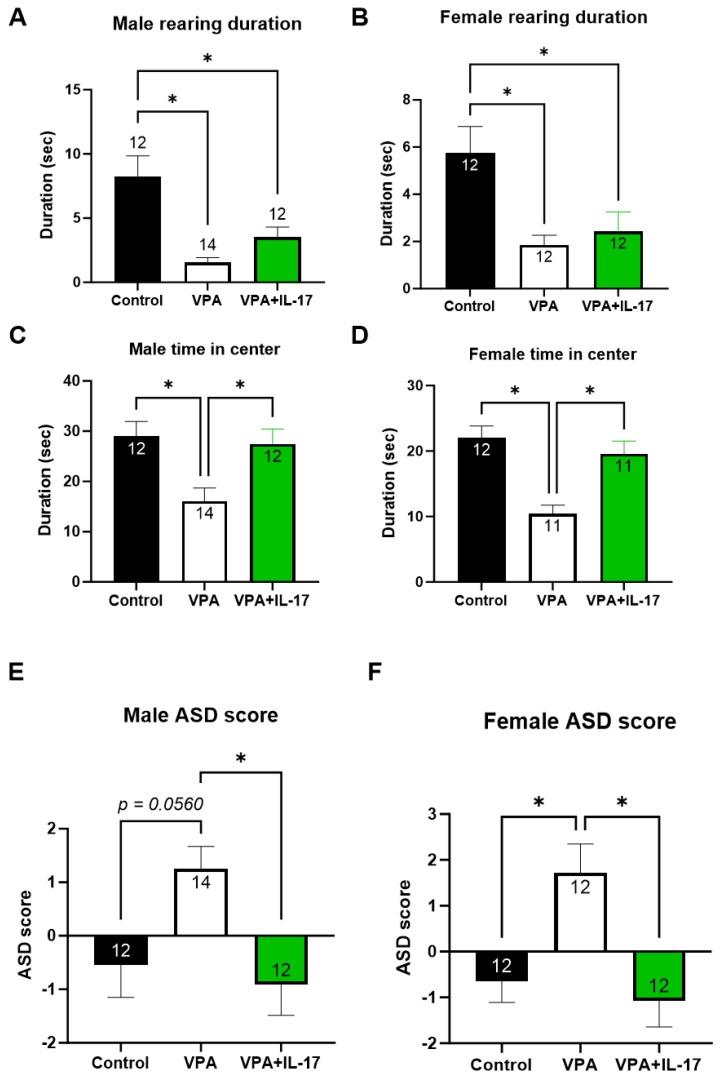
Administration of IL-17A reduced the ASD-like phenotype in VPA-exposed mice. Anxiety-related behavior was measured by rearing duration (**A**,**B**) and total duration in the center of the open field arena (**C**,**D**) for males and females, respectively. Assessment of the general ASD-like phenotype was performed for each mouse by summarizing z-standardized scores for social interaction, water T-maze, rearing, and time in center as measured in the open field paradigm. Higher scores correspond with an increased ASD-like phenotype in males (**E**) and females (**F**). Control_represents control mice group, not exposed to VPA and without any treatment. VPA_represents mice exposed to VPA modelling ASD without any treatment. VPA+IL-17_represents mice exposed to VPA that were treated with IL-17A at adulthood. Data are presented as the mean ± SE and the number of samples per group is presented within the bars. * *p* < 0.05, one-way ANOVA. No significant difference between the sexes was observed in two-way ANOVA.

In the open field paradigm, no significant differences were observed between the different groups in general locomotion activity and grooming activity. However, reduced rearing activity, reflecting increased anxiety, was noted in VPA-exposed mice w/o IL-17A treatment compared with controls in both sexes (Figure 3A,B). Similarly, a reduced duration in the center of the area, reflecting increased anxiety, was noted in VPA-exposed mice. However, here, IL-17A treatment normalized the duration time in VPA-exposed male and female mice (Figure 3C,D). When we combined all the above measurements into one score per mouse representing each mouse level of ASD-like phenotype (henceforth ASD score), we found a significant increase in the score of VPA-exposed mice compared with controls and IL-17A-treated animals in both sexes (Figure 3E,F).

### 2.2. Altered Hippocampal Neurogenesis following Developmental Exposure to VPA Is Partly Corrected by IL-17A Treatment

**Figure 4:**
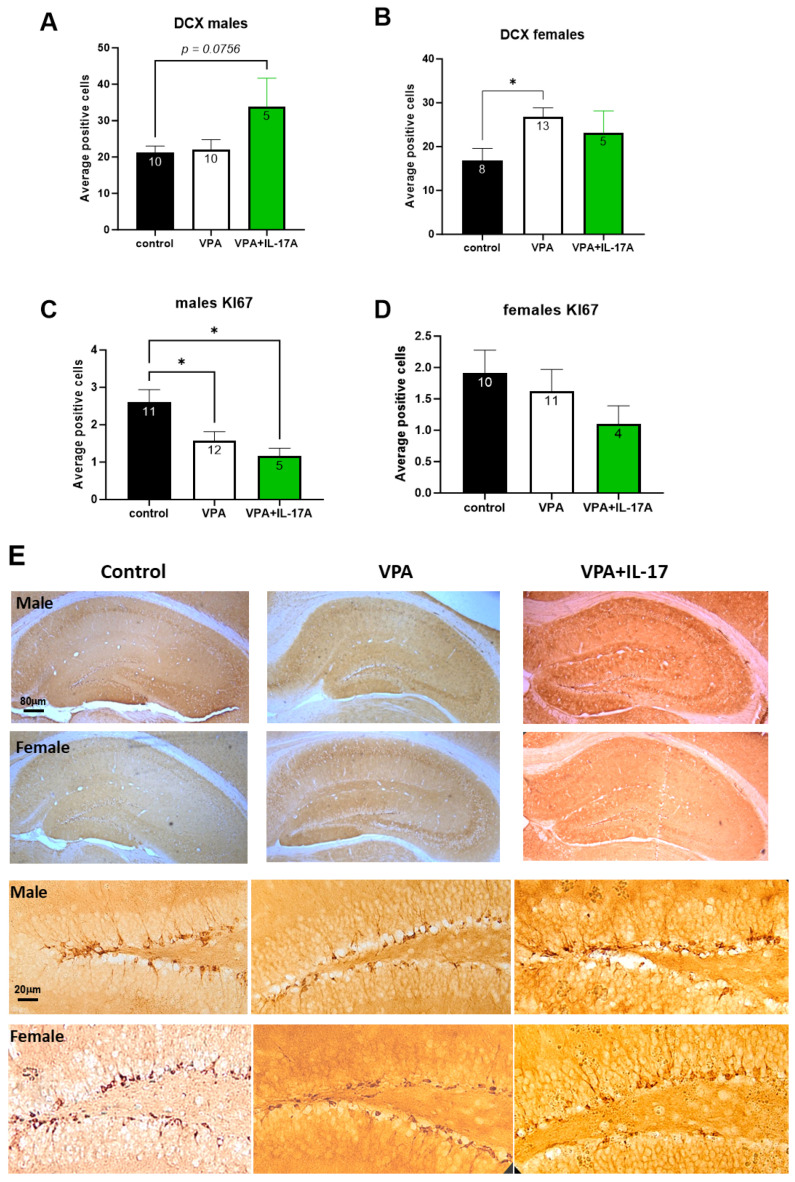
IL-17A partly corrected the altered adult hippocampal neurogenesis resulting from developmental exposure to VPA. Hippocampal neurogenesis was assessed by immunohistochemistry for early differentiating neurons expressing doublecortin (DCX) in the granular cell layer (GCL) and proliferating neuroprogenitors (positive for Ki67) in the subgranular zone of the dentate gyrus. (**A**) Graph presenting the average DCX^+^ cells in the GCL per hippocampal section in males. (**B**) Graph presenting the average DCX^+^ cells in the GCL per hippocampal section in females. (**C**) A graph presenting the average positive Ki67 nuclei in the subgranular zone per hippocampal section in males. (**D**) A graph presenting the average positive Ki67 nuclei in the subgranular zone per hippocampal section in females. (**E**) Low- and high-power micrographs of DCX^+^ cells in the GCL. Control—represents control mice group, not exposed to VPA and without any treatment. VPA—represents mice exposed to VPA modelling ASD without any treatment. VPA+IL-17—represents mice exposed to VPA that were treated with IL-17A at adulthood. Data are presented as the mean ± SE and the number of samples per group is presented within the bars. * *p* < 0.05, one-way ANOVA. No significant difference between the sexes was observed in two-way ANOVA.

Animals were sacrificed two weeks following IL-17A treatment and behavioral tests. Analysis was performed on all treatment groups (control, VPA, and VPA+IL-17A). Brains were removed and assessed for hippocampal neurogenesis. The assessment of neurogenesis in the adult hippocampus was performed by immunohistochemistry (IHC) staining targeting doublecortin (DCX), which is expressed in newly formed neurons in the granular cell layer (GCL) of the dentate gyrus (DG), and Ki67, a marker for cell proliferation in early progenitor cells in the subgranular zone (SGZ). VPA-exposed mice displayed sex-dependent alterations in neurogenesis. While in males, the number of Ki67^+^ cells in the SGZ was reduced in VPA-exposed mice, in females, an increased number of DCX^+^ cells in the GCL was observed (Figure 4). IL-17A treatment did not reverse these changes; however, in males, a close to significant increase in DCX^+^ cells was observed in IL-17A-treated animals (Figure 4A).

**Figure 5:**
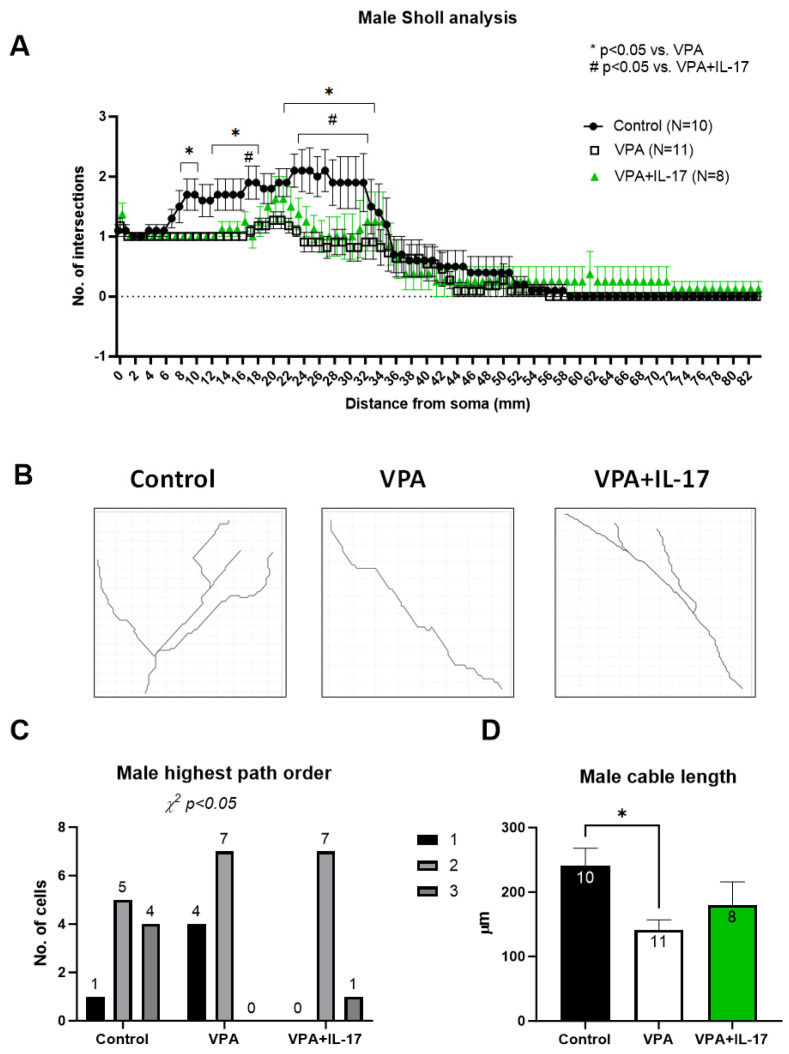
IL-17A slightly improved neuronal development impairment resulting from developmental exposure to VPA in males. Newly formed neurons expressing doublecortin in the granular cell layer (GCL) of male animals were analyzed for neurite growth and complexity in their dendritic tree following immunohistochemistry for doublecortin (DCX). (**A**) Sholl analysis performed on newly formed DCX+ neurons in the GCL. The graph demonstrates the average number of intersections with respect to the distance from the cell soma. (**B**) Representative neurite branching paths in the different treatment groups. (**C**) Strahler highest path order distribution analysis in the different groups. Significance of distribution differences was performed by Chi-squared analysis. (**D**) A graph presenting the average total neurite length (cable length) in the different treatment groups. Control_represents control mice group, not exposed to VPA and without any treatment. VPA_represents mice exposed to VPA modelling ASD without any treatment. VPA+IL-17_represents mice exposed to VPA that were treated with IL-17A at adulthood. Data are presented as the mean ± SE and the number of samples per group is presented within the bars. ^#,^* *p* < 0.05. two-way ANOVA for (**A**) and one way ANOVA for (**D**).

**Figure 6:**
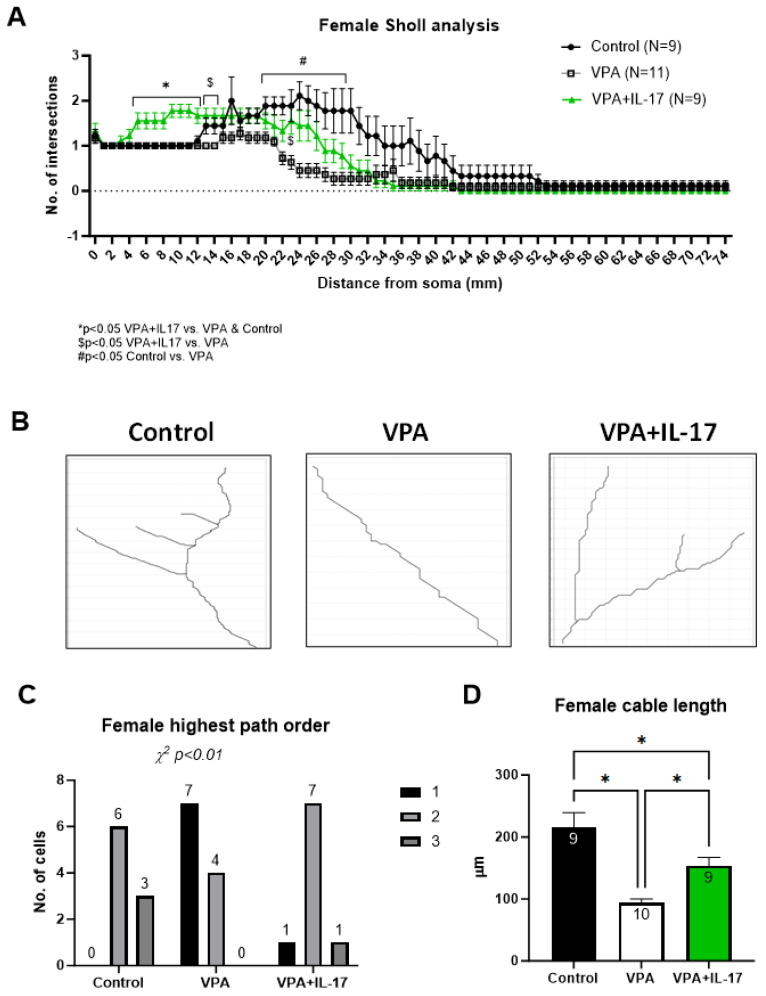
IL-17A improved neuronal development impairment resulting from developmental exposure to VPA in females. Newly formed neurons expressing doublecortin in the granular cell layer (GCL) of male animals were analyzed for neurite growth and complexity in their dendritic tree following immunohistochemistry for doublecortin (DCX). (**A**) Sholl analysis performed on newly formed DCX+ neurons in the GCL. The graph demonstrates the average number of intersections with respect to the distance from the cell soma. (**B**) Representative neurite branching paths in the different treatment groups. (**C**) Strahler highest path order distribution analysis in the different groups. Significance of distribution differences was performed by Chi-squared analysis. (**D**) A graph presenting the average total neurite length (cable length) in the different treatment groups. Control_represents control mice group, not exposed to VPA and without any treatment. VPA_represents mice exposed to VPA modelling ASD without any treatment. VPA+IL-17_represents mice exposed to VPA that were treated with IL-17A at adulthood. Data are presented as the mean ± SE and the number of samples per group is presented within the bars. ^#,$,^* *p* < 0.05, two-way ANOVA for (**A**) and one way ANOVA for (**D**).

Although an increase in DCX^+^ cells was noted in VPA-exposed females, a distinct morphological difference in these cells was noted as well. In both male and female VPA-exposed animals, the dendritic tree of DCX^+^ cells was shorter and less complex than that of cells in the control group. Sholl and Strahler analysis demonstrated decreased neurite tree complexity in VPA-exposed mice (Figure 5A–C and Figure 6A–C), and decreased total length was also observed in SNT (Simple Neurite Tracing) analysis (Figure 5D and Figure 6D). No significant differences were observed between the sexes in two-way ANOVA. IL-17A treatment partly corrected these deficiencies, notably in females (Figure 5 and Figure 6).

### 2.3. Treatment with IL-17A Induced Changes in Serum Levels of T-Helper-Related Cytokines in Animals Developmentally Exposed to VPA

**Figure 7:**
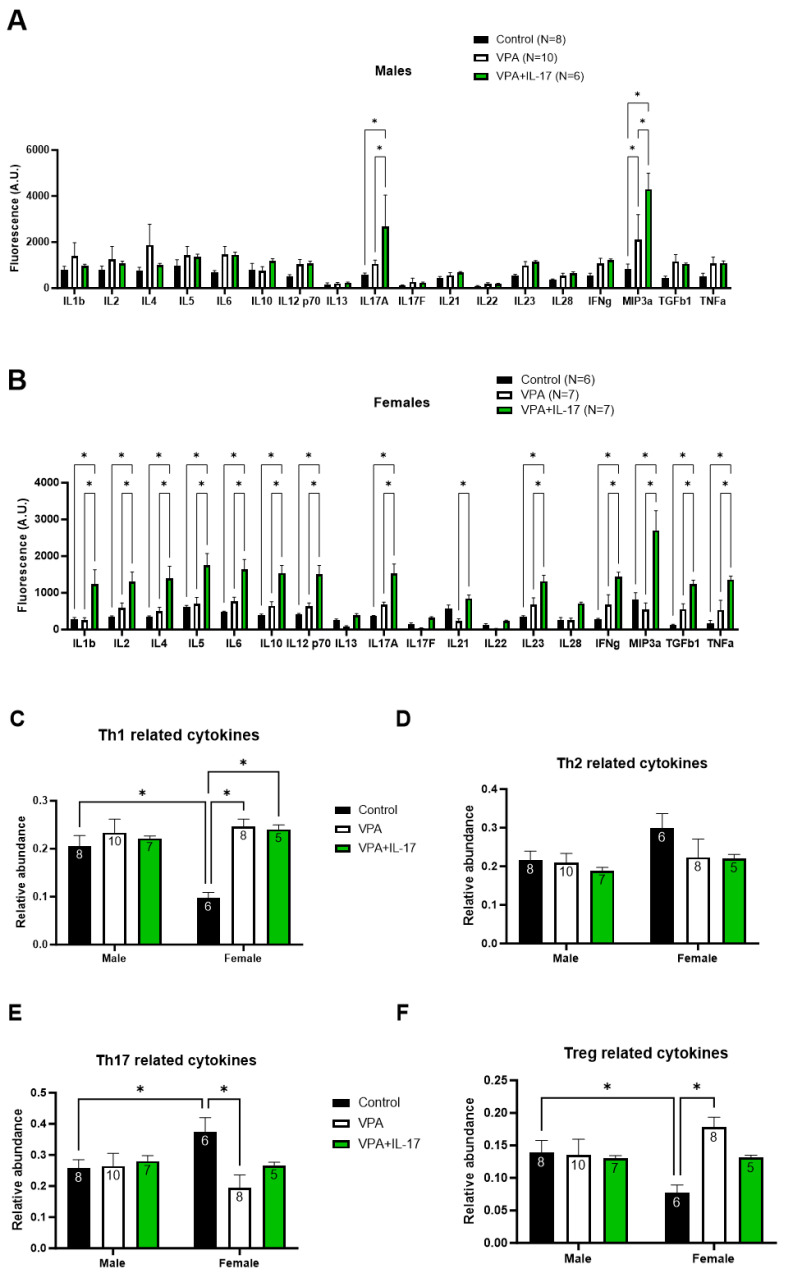
Developmental VPA exposure and IL-17A treatment alter the serum T-helper-related cytokine profile. Serum collected from mice at the end of the experiments (two weeks after IL-17A administration) was analyzed for the levels of T-helper-related cytokines using a semiquantitative antibody array. (**A**,**B**) Graphs presenting the average serum levels of the tested cytokines in males and females. The results are presented as average fluorescence intensity in arbitrary units. The relative abundance of groups of cytokines was calculated and is presented in the following graphs depicting Th1-related cytokines (**C**), Th2-related cytokines (**D**), Th17-related cytokines (**E**), and Treg-related cytokines (**F**). Control_represents control mice group, not exposed to VPA and without any treatment. VPA_represents mice exposed to VPA modelling ASD without any treatment. VPA+IL-17_represents mice exposed to VPA that were treated with IL-17A at adulthood. Data are presented as the mean ± SE and the number of samples per group is presented within the bars. * *p* < 0.05, two-way ANOVA.

Two weeks following treatment with IL-17A and subsequent behavioral testing, animals were sacrificed, and serum was collected and analyzed for the levels of T-helper-related cytokines using a Quantibody array. In both males and females, exogenous treatment with IL-17A increased endogenous serum levels of IL-17A (Figure 7A,B). However, while in males, only IL-17A and MIP-3α were increased following IL-17A treatment, in females, multiple cytokines were increased, including IL-1β, IL-2, IL-4, IL-5, IL-6, IL-10, IL-12p70, IL-17A, IL-21, IL-23, IFN-γ, MIP-3α, TGF-β1, and TNF-α. To compare groups of specific T-helper cytokines, we first normalized each cytokine level (min–max) and calculated its relative abundance in each serum sample. Then, we summarized the relative abundance of specific groups of T-helper-related cytokines accordingly: Th1 (IL-1β, IL-12p70, IFN-γ, TNF-α), Th2 (IL-4, IL-5, IL-6, IL-13), Th17 (IL-17A, IL-17F, IL-21, IL-22, IL-23), and Treg (IL-10, TGF-β1). In males, we did not observe any significant changes in Th-related cytokines between the different groups (Figure 7C–F). However, changes were observed in females. Th1 and Treg-related cytokines were significantly increased in VPA-exposed females compared with control females (Figure 7C,F). IL-17A treatment slightly decreased Treg-related cytokines and did not significantly differ from controls (Figure 7F). An additional sex-dependent difference was noted, as female controls had significantly lowered relative abundances of Th1- and Treg-related cytokines compared with male controls. An opposite observation was noted with Th17-related cytokines, as VPA developmental exposure significantly reduced their relative abundance. Control females presented a significant increase in the relative abundance of Th17-related cytokines compared with males (Figure 7E).

**Figure 8:**
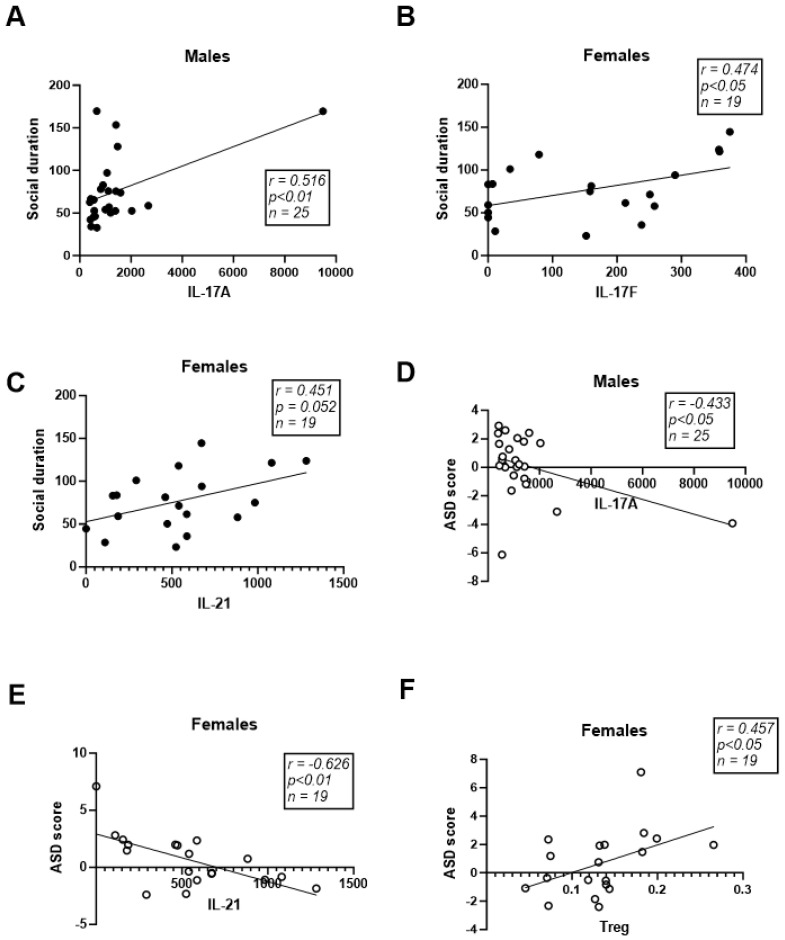
Serum cytokine levels are associated with ASDlike behavior. Scatter plots and their corresponding regression plots, representing the correlation between social interaction duration and cytokine levels (**A**–**C**). Scatter plots and their corresponding regression plots, representing the correlation between ASD score and cytokine levels (**D**,**E**). Scatter plot and its corresponding regression plot, representing the correlation between ASD score and Tregrelated cytokine relative abundance in females (**F**). Pearson correlation.

Using Pearson correlation analysis, we assessed the association between serum cytokine levels and ASD-like behavior. We found social behavior (social interaction duration) to be positively correlated with IL-17A serum levels in males and with IL-17F and IL-21 in females (Figure 8A–C). The ASD score was negatively correlated with IL-17A levels in males and with IL-21 levels in females (Figure 8D,E). Furthermore, the ASD score was positively correlated with the relative abundance of Treg-related cytokines in females (Figure 8F).

## 3. Discussion

IL-17, as a proinflammatory cytokine, was suggested as a possible etiology for neurodevelopmental disorders such as ASD [35,36]. It was shown that developmental exposure to maternal immune activation was associated with social behavior impairment in adulthood in mice [13,37]. Surprisingly, however, intracerebral treatment with IL-17A was able to mitigate social impairment, suggesting a positive role for the cytokine in modulating social behavior [13,38]. Similarly, we previously demonstrated that elevated serum levels of IL-17 were associated with increased sociability in mice exposed to trauma [32] and with increased social novelty preference in mice with schizophrenia [39]. Elevated blood levels of IL-17 and Th17 were also associated with improved cognitive functions in schizophrenia patients following treatment [40]. In the present study, we observed improved social behavior and reduced overall ASD-like behavior following two injections of the cytokine IL-17A (Figure 1 and Figure 3E,F). We found that serum levels of Th17-related cytokines, i.e., IL-17A in males and IL-17F and IL-21 in females, were positively correlated with social behavior and negatively correlated with the total ASD score (Figure 8). Interestingly, two IL-17A intravenous treatments caused elevated serum levels of IL-17A two weeks later both in males and females (Figure 7A,B), although the estimated half-life for IL-17A is 8 h [41]. We postulate that IL-17A treatment elicited a positive feedback loop elevating endogenous expression of IL-17A, as previously suggested [42,43].

Modulation of the immune system toward a pro-inflammatory phenotype was previously suggested as a key feature in ASD patients [44]. Children with ASD with gastrointestinal (GI) comorbidities exhibited elevated populations of peripheral Th17 cells compared with ASD children without GI comorbidities, whereas ASD children without GI comorbidities were characterized by increased Th2 and Th1 populations compared with normal children [45]. More recently, elevated levels of Th1, Th2, and Th17 subpopulations and decreased levels of Treg were reported in ASD-diagnosed children compared with control children [46]. In the VPA model for ASD in mice, an increased inflammatory response with evidence for neuroinflammation was also observed in males, suggesting similar deregulation of the immune system in the VPA model as well [47]. In the present study, we found no significant differences in serum cytokine levels between VPA-exposed animals and controls in either males or females (Figure 7A,B); nevertheless, when comparing the relative abundance of Th-related cytokines, sex-dependent differences were observed (Figure 7C–F). While males displayed no differences between the different cytokine groups, in females, the relative abundance of Th1- and Treg-related cytokines was higher in VPA-exposed animals than in controls, and vice versa for Th17-related cytokines. IL-17A treatment partly corrected the changes observed following VPA exposure. Interestingly, the relative abundance of these Th-related cytokine groups was significantly different between control males and females, with control males presenting abundance levels resembling those of VPA-exposed females. IL-17A treatment also increased the serum levels of multiple cytokines in females compared with only two cytokines in males, suggesting a baseline difference in immune function between males and females in ASD, as was previously hypothesized [48]. These differences correspond with our previously reported sex-dependent differences in behavior and prefrontal cortex gene expression in mice developmentally exposed to VPA modeling ASD [33,34].

The effect of IL-17A on social behavior was previously associated with reduced neuronal activation in the somatosensory cortex (S1DZ), as observed in maternal immune activation-affected mouse offspring [13]. In the present study, we sought to explore the possible effect of IL-17A on adult hippocampal neurogenesis. In previous studies, we and others have shown impaired adult hippocampal neurogenesis in mice prenatally exposed to VPA [26,27]. Reduced neuroprogenitor proliferation, reduced neuronal maturation, reduced neurite length, and aberrant morphology in the dentate gyrus were reported in these studies. Similarly, in the present study, we found that males had a significantly reduced number of proliferating neuroprogenitors in the subgranular zone in the dentate gyrus, with a similar but not significant trend in females (Figure 4C,D). However, females exposed to VPA displayed an increased number of doublecortin (DCX)-expressing newly formed neurons compared to controls (Figure B). Nevertheless, in both males and females, DCX^+^ newly formed neurons had an aberrant morphology with decreased neurite length and dendritic tree complexity (Figure 5 and Figure 6). Impaired neuritogenesis of hippocampal neurons was also reported previously in the BTBR mouse model of ASD [49]. Impaired neuritogenesis corresponds with aberrant synaptogenesis observed in ASD patients [24,50]. Treatment with IL-17A did not increase neuroprogenitors’ proliferation in males or in females (Figure 4C,D), as previously reported [31,32,51]. However, IL-17A treatment promoted DCX^+^ cell neuritogenesis (Figure 5 and Figure 6), in accordance with previous findings [31]. Similarly, human neuroprogenitors derived from induced pluripotent stem cells obtained from ASD and control patients displayed increased neuronal differentiation and synaptogenesis in vitro in response to exogenous IL-17A [52].

In conclusion, although IL-17A can play a key role in the immune-related pathogenesis of ASD during brain development, our study shows that it can also play a role in preserving the behavioral phenotype at a later age. These results correspond with the report of Reed et al., demonstrating increased social behavior following intracerebral administration of IL-17A in a neurodevelopmental model of social deficits induced by maternal immune activation [13]. Surprisingly, a similar phenomenon was also observed in a model of traumatic brain injury, where IL-17A KO mice performed worse in a memory retention task following injury than control mice [53]. Thus, we suggest a potential dual role for IL-17 in ASD, both as a mediator of its pathology and as a key element in resolving its behavioral deficits. We propose that IL-17′s effect on neurogenesis and particularly on neuritogenesis is an important mechanism in alleviating ASD-like behavior.

## 4. Materials and Methods

### 4.1. Animals

All experimental procedures were approved by Ariel University’s Animal Care and Use Committee and were performed in accordance with National Institutes of Health guidelines. Pregnant female ICR mice were purchased from Envigo (Jerusalem, Israel) and housed at a temperature of 22 °C under a 12:12 h dark–light cycle. Food (Teklad Global Diet from Harlan Laboratories Inc., Jerusalem, Israel) and water were provided ad libitum. Dams were housed with their mothers until weaning on post-natal day 21. After weaning, mice were separated by sex and housed in 36.5 cm × 20.7 cm × 14 cm cages (5 animals/cage) with appropriate bedding and paper rolls as enrichment. Experimental design (Figure 1A): At post-natal day 4, neonatal pups, at an average wight of 3 g, were subcutaneously injected with a single dose of 300 mg/kg VPA (cat. No. P4543-10G, Sigma Aldrich, St. Louis, MO, USA) dissolved in 0.9% saline solution. At the age of seven weeks, at an average weight of 35 g, offspring were intravenously injected twice (three days apart) with 8 µg of recombinant murine IL-17A (cat. No. 210-17-500, PeproTech, Cranbury, NJ, USA) dissolved in 0.9% saline solution, into the tail vein. Control offspring were injected with vehicle. Animals were randomly assigned to experimental groups. Behavioral assays were conducted in weeks 8 and 9. Following behavioral assays, mice were anesthetized with a ketamine (150 mg/kg) xylazine (10 mg/kg) mixture, diluted in 0.9% saline, and approximately 800 µL of blood was collected directly from the heart. Mice were then sacrificed by intracardial perfusion with saline followed by 4% paraformaldehyde (PFA) (cat. No. 47608-1L-F Sigma Aldrich, St. Louis, MO, USA). 

### 4.2. Behavioral Assays

To assess ASD-like behavior, we applied a series of behavioral paradigms as we previously described and included open field, three-chamber social interaction, and water T-maze [32,33]. Experiments were conducted from early morning to noon time (corresponding with the light cycle of the animals). Detailed description of the paradigms is provided in the following subsections. 

#### 4.2.1. Three-Chamber Social Interaction

Animal sociability was tested using the three-chamber social interaction paradigm [32,33]. The arena is composed of three chambers, with opposing chambers containing small cages. During the first phase, each mouse was inserted for ten minutes into the paradigm arena (80 cm × 27 cm) for habituation. Thirty minutes later, the tested animal was returned to the arena when one of the two cages in the arena was occupied by an unfamiliar mouse (social stimulus) and the other cage remained empty. During the 10 min test, the time spent exploring the social stimulus versus the empty cage was recorded using a computerized video system and software EthoVision version 16 (Noldus; Wageningen, The Netherlands). The arena was cleaned with 70% ethanol between tests.

#### 4.2.2. Open Field Test

The open field test was used to assess locomotor behavior [32,33]. Mice were placed near the midpoint of a black plastic box measuring 40 × 40 × 40 cm for a period of 5 min. Mice were naïve to the area and did not have any previous encounter with it. Using a computerized video system and software, the total distance that the animal walked and the time spent in the center of the arena were measured. The total duration of rearing behavior (standing without support on the lower limbs) was manually measured. The arena was cleaned with 70% ethanol between each animal.

#### 4.2.3. Water T-Maze

Repetitive behavior and cognitive rigidity were assessed using the water T-maze paradigm [33]. In this test, the mice were inserted into the base of a T-shaped water bath (25 °C) with a hidden platform on one of the T-maze arms. On the first two days of the assay, the platform was placed on the left arm of the maze, and the animals learned the location of the platform in ten trials per day. Then, on the third day, the platform was moved to the right arm of the maze, and the time to relocate the platform was measured over ten trials. Adaptation to the platform change was demonstrated by decreased latency duration on day 3. The average latency of day 3 was calculated and used for calculating the ASD score.

#### 4.2.4. ASD Score Calculation

A combined ASD score was calculated as previously described [33,54]. Briefly, the results of each behavioral assay were standardized using Z-standardization. The Z scores from all parameters were totaled for each animal, in a way that a positive score corresponds with increased ASD-like behavior; namely, Z-scores for the water T-maze (day 3 average latency) were added and the Z-scores for sociability (time with social stimulus), time in center, and rearing duration in the open field were subtracted as depicted in the formula:

ASD score = T-maze − Social interaction − Time in center − Rearing duration.

### 4.3. Th1/Th2/Th17 Array

Following behavioral assays, mice were anesthetized with a ketamine (150 mg/kg) xylazine (10 mg/kg) mixture and approximately 800 µL of blood was collected directly from the heart, incubated for 30 min at room temperature in plastic tube serum, and harvested following blood coagulation. To assess the levels of Th1/Th2/Th17 immune mediators, an array of semiquantitative detectors was applied according to the manufacturer’s protocol (cat. No. GSM-TH17-1-1, Raybiotech, Peachtree Corners, GA, USA) [55]. All reagents were supplied by the manufacturer. Briefly, 100 µL of Sample Diluent buffer was added into each well at RT for 30 min to block slides. Buffer was decanted from all wells, 50 µL of serum and 50 µL of Sample Diluent buffer were added, and the array was incubated overnight at 4 °C. Samples were decanted and wells were washed five times with 150 µL of Wash Buffer I, followed by two washes with Wash Buffer II at room temperature with gentle shaking. Eighty microliters of the detection antibody cocktail were added to each well and incubated at room temperature for two hours. Following incubation, the antibody cocktail was decanted and the wells were washed as previously described. Then, 80 µL of Cy3 equivalent dye-conjugated streptavidin was added to each well and incubated for one hour at room temperature in the dark. After incubation, the wells were washed in the same manner as previously described, and after completely drying, the signals were visualized using a laser scanner equipped with a Cy3 wavelength provided by the manufacturer.

To assess the relative abundance of Th-related cytokines, the fluorescence signals for each cytokine were standardized using min–max standardization [56]. The standardized score for each cytokine was calculated for its relative abundance by calculating the ratio between each cytokine score and the total of all cytokine scores for each animal. Th-related cytokine relative abundance was calculated by summing the relative abundance of all cytokines related to a particular T-helper as follows: Th1 (IL-1β, IL-12p70, IFN-γ, TNF-α), Th2 (IL-4, IL-5, IL-6, IL-13), Th17 (IL-17A, IL-17F, IL-21, IL-22, IL-23), and Treg (IL-10, TGF-β1).

### 4.4. Immunohistochemical Staining

Immunohistochemistry staining was performed to assess hippocampal neurogenesis as previously described [32,39]. Following behavioral assays, mice were sacrificed by intracardial perfusion with saline followed by 4% paraformaldehyde (PFA) (cat. No. 47608-1L-F Sigma, Jerusalem, IL). The brains were removed, postfixed overnight, and equilibrated in phosphate-buffered 30% sucrose solution. Frozen brain tissue sections (20 μm) were prepared using a semi-automatic cryostat (SLEE Medical GmbH, Niederolm, Germany). Hippocampal sections were stained for the neuronal differentiation marker doublecortin (DCX) (cat. No. ab18723 Abcam, Cambridge, UK) and the proliferation marker Ki-67 (cat. No. ab15580 Abcam, Cambridge, UK) using an immunohistochemistry kit according to the manufacturer’s protocol (cat. No. MP-7451 Vector laboratories, Newark, CA, USA). The membrane permeabilization step was performed using 2% Triton X-100 for five minutes, followed by incubation for 10 min with 3% H_2_O_2_ to block endogenous peroxidase, followed by incubation with blocking solution (provided by the manufacturer) for 45 min at room temperature. Sections were then incubated with primary rabbit polyclonal anti-DCX (diluted 1:500 in PBS) or primary rabbit polyclonal anti-Ki-67 (diluted 1:500 in PBS) overnight at 4 °C. The next day, the sections were incubated with horseradish peroxidase (HRP) one-step polymer-conjugated secondary antibody for 1 h at room temperature. Visualization was achieved with 5 min incubation with 3,3’ diaminobenzidine tetrahydrochloride buffer (cat. No. VE-SK-4105 Vector laboratories, Newark, CA, USA). Between steps, sections were washed three times with PBS for five minutes. For the quantification of DCX- and Ki-67-positive cells and nuclei in the granular cell layer and subgranular zone, respectively, at least eight representative sections were counted for each mouse, and an average was calculated. Micrographs were acquired using an OLYMPUS BX53 microscope (Tokyo, Japan) equipped with an OLYMPUS camera U-TV0.5XC-3 with OLYMPUS CellSens imaging software (Version 1.18).

Neurite morphology, including cable length, complexity, and Sholl analysis for DCX^+^ cells, was performed using the SNT and Sholl analysis modules of FIJI-ImageJ software (Version 1.54f) [57]. For the analysis, approximately 10 cells originating from three different animals were analyzed for each treatment group (i.e., Control, VPA, and VPA+IL-17).

### 4.5. Statistical Analysis

All data in graphs are presented as the mean with bars representing standard error. Data were assessed for normal distribution using the Shapiro-Wilk and Kolmogorov-Smirnov tests with a significance level of α = 0.05. Outliers were detected using Grubb’s test with α = 0.05. Multiple comparisons between more than two groups were assessed for significance using one-way and two-way analysis of variance (ANOVA) followed by Tukey’s post hoc test. One-way ANOVA was applied when comparing multiple groups according to only one independent categorial variable (i.e., treatment groups). Two-way ANOVA was applied when additional independent variable was added to the analysis (i.e., sex, different time points or social/nonsocial categories). Significance between each two groups was determined with 95% confidence interval, α = 0.05 (*p* < 0.05). Every possible comparison between the study groups was considered. Correlations were calculated using the Pearson correlation test. Differences in distribution of categorical variables were calculated using chi-squared analysis. Statistical analysis and graphs were performed using Prism GarphPad version 10.1.0. 

## Data Availability

The data that support the results of this study are available from the corresponding author of the article upon reasonable request.
